# Transient White Matter Diffusion Restriction in a Pediatric Patient With Diffuse Axonal Injury

**DOI:** 10.7759/cureus.116362

**Published:** 2026-09-16

**Authors:** Claudia Lazarte-Rantes, Carol Munayco, Mario Chirinos

**Affiliations:** 1 Radiology, Instituto Nacional de Salud del Niño San Borja, Lima, PER; 2 Radiology, Universidad Nacional Mayor de San Marcos, Lima, PER; 3 Neurology/Pediatric Neurology, Instituto Nacional de Salud del Niño San Borja, Lima, PER

**Keywords:** cytotoxic edema, diffuse axonal injury, pediatric, transient diffusion restriction, traumatic brain injury

## Abstract

Traumatic brain injury (TBI) is a serious public health problem due to its high incidence worldwide and possible sequelae, especially in the pediatric population. Imaging studies play a leading role in evaluating pathologies requiring early surgical management and the prognosis of long-term sequelae. Diffuse axonal injury (DAI) is one of the most severe complications of TBI, and few reports in the literature are associated with cytotoxic edema. We present a case of a pediatric patient with DAI with extensive areas of transient diffusion restriction in the white matter, which we consider to be related to cytotoxic edema.

## Introduction

Traumatic brain injury is defined as the alteration of brain function or evidence of other brain pathology caused by the application of an external force [1].

Recent studies [2] show a worldwide incidence of 939 new cases per 100,000 inhabitants per year, with a regional incidence in Latin America of 909 per 100,000. In Peru, studies indicate that TBI occurs in 57% of trauma cases in the pediatric population. The principal cause was falls, and the second cause was a motor vehicle accident [3].

The lesions in the brain are related to mechanical injury that predisposes to the appearance of primary lesions (produced by the initial mechanical forces) and secondary lesions (caused by edema, hypoxia, etc.) [4]. Pediatric patients who present with TBI have a high morbidity with multiple motor and cognitive sequelae [5]. Recent studies show a direct correlation between the presence of certain imaging findings and the prognosis in the rehabilitation of these patients, giving greater emphasis to the early use of imaging studies [5].

Diffuse axonal injury (DAI) in TBI is produced by a direct mechanical lesion of axonal fibers with subsequent apoptotic and neurodegenerative effects [6]. Few case reports indicate that patients with DAI can present cytotoxic edema that, in some cases, has been associated with diffusion restriction [7].

## Case presentation

A 10-year-old female with no significant medical history had a motor vehicle accident. She was admitted to the emergency department with signs of hypoperfusion, multiple excoriations, otorrhagia, and epistaxis. She was admitted to the pediatric intensive care unit (PICU) with the diagnoses of severe traumatic brain injury, hypovolemic shock, pneumothorax, and coagulation disorder. On admission, she had a score of eight on the Glasgow Coma Scale and slow photoreactive isochoric pupils. She was intubated and was under sedation with a midazolam infusion. She presented with electrolyte disorders wherein her sodium (Na) was at 150 mEq/L, potassium (K) was at 2.5 mEq/L, and chlorine (Cl) was at 126 mEq/L, as well as severe anemia with a hemoglobin (Hb) of 6.6 g/dL.

A tomographic study was performed on admission (approximately four hours after the accident), which showed multiple hyperdense punctate areas initially classified as areas of hemorrhagic contusion of cortico-subcortical localization and deep white matter of the right fronto-parietal-occipital and right frontotemporal lobes. Hemorrhage in the right cerebellar hemisphere and the fourth ventricle was observed (Figure 1).

**Figure 1:**
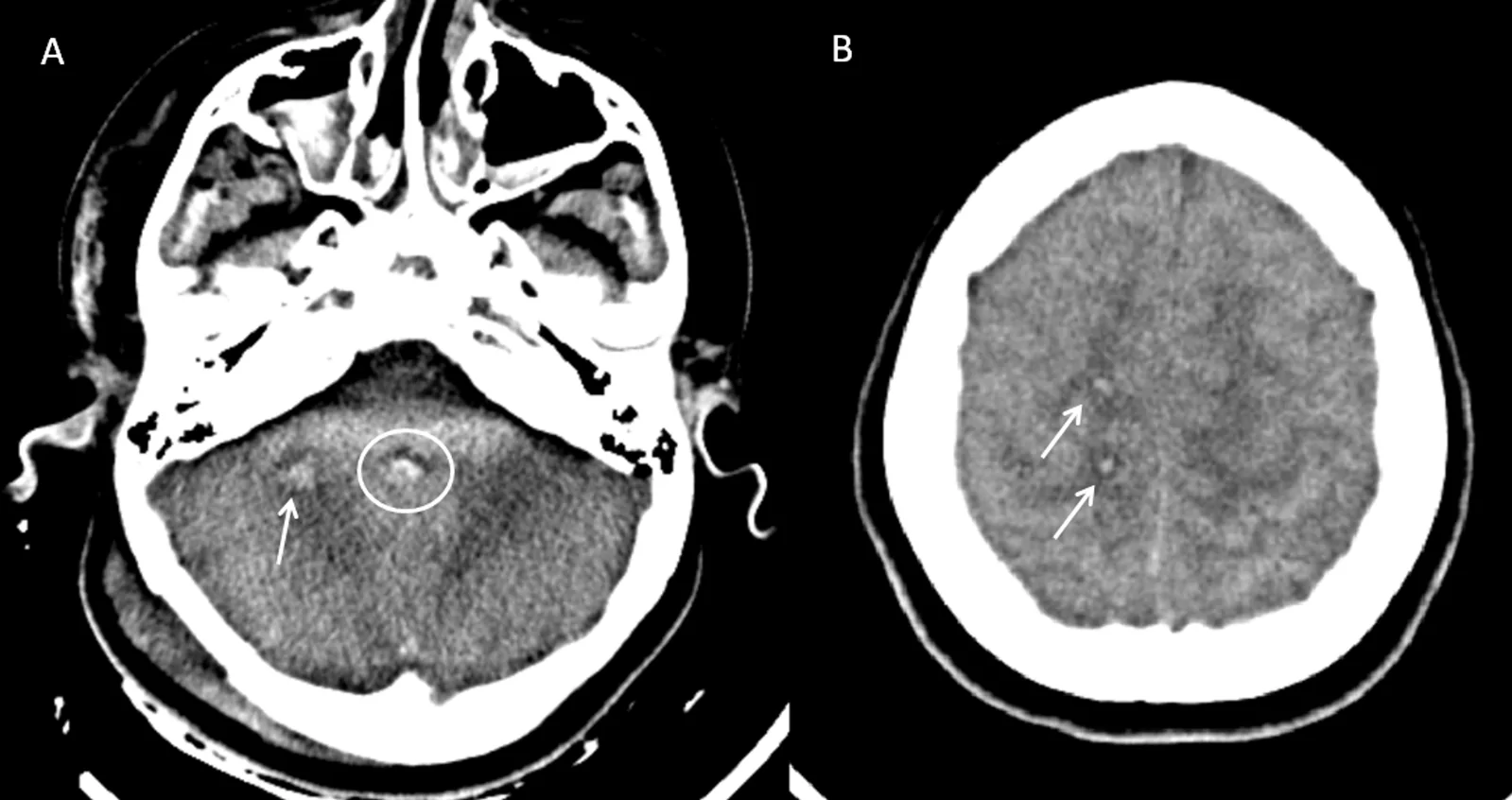
The first brain CT taken at admission An axial CT head image at the level of the posterior fossa (A) shows areas of high density in the right cerebellar lobe (arrow) and fourth ventricle (circle) compatible with hemorrhages. Other areas of hemorrhages and the level of white matter (B)

Additionally, multiple maxillofacial fractures and right temporal fractures were observed. Chest and abdominal CT scans revealed pneumothorax, hepatic contusion, and right renal contusion (not shown).

Seven days after her admission, a brain MRI was performed without contrast, showing extensive confluent hyperintense areas in the T2 weighted image (T2WI) and fluid-attenuated inversion recovery (FLAIR) in the deep white matter of bilateral fronto-parieto-occipital lobes with extension to the corpus callosum and right temporal region, and punctate areas with susceptibility artifacts in the susceptibility-weighted imaging (SWI) were observed in both cerebral hemispheres. Extensive areas of diffusion restriction were also observed in the corpus callosum and white matter of both cerebral hemispheres (Figure 2).

**Figure 2:**
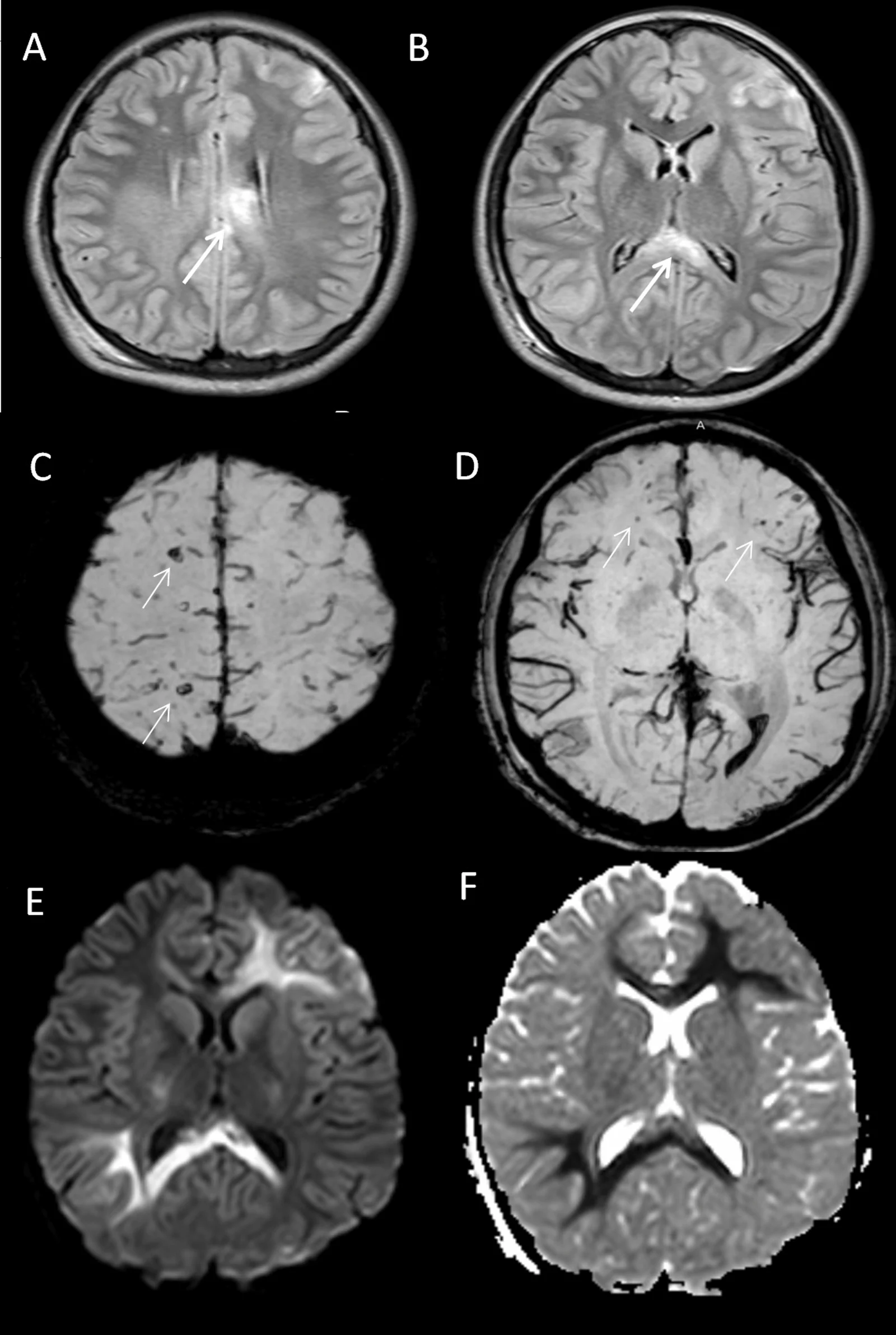
Brain MRI done seven days after admission to the emergency room Axial FLAIR images (A and B) show bilateral asymmetric hyperintensities in deep white matter and the body of the corpus callosum (arrow). Axial SWI (C and D) show punctate areas with susceptibility artifacts in the white matter of both cerebral hemispheres (arrows) and corpus callosum (not shown), in keeping with DAI. Additionally, the axial diffusion and ADC maps (E and F) demonstrate extensive areas of diffusion restriction in the white matter. FLAIR: fluid-attenuated inversion recovery; SWI:susceptibility-weighted imaging; DAI: diffuse axonal injury; ADC: apparent diffusion coefficient

Additionally, there was an area of bleeding in the right cerebellar lobe and subdural hematomas in the occipital region. A control MRI study was performed 10 days after admission, showing complete resolution of the restriction diffusion seen before (Figure 3).

**Figure 3:**
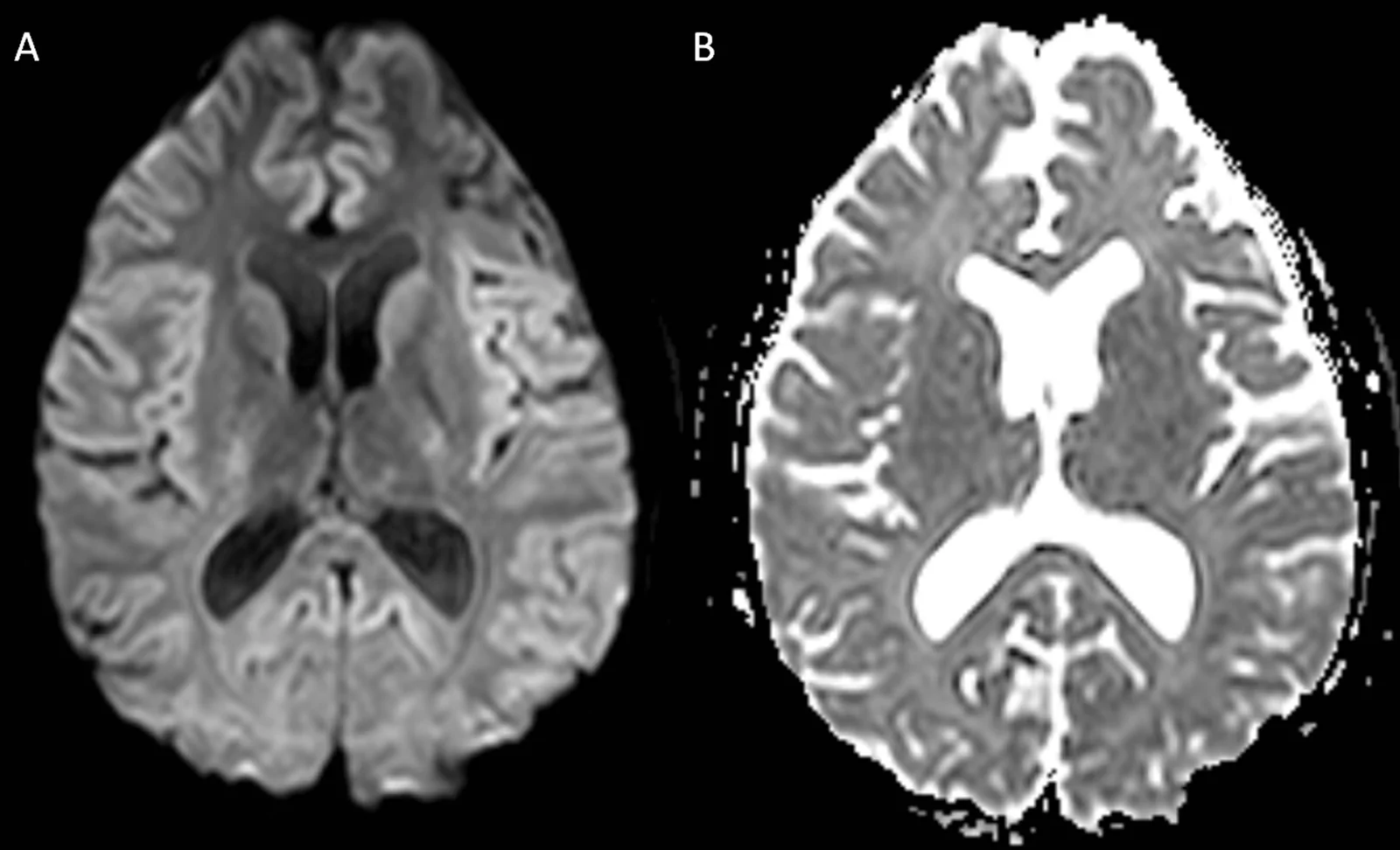
Control brain MRI done 10 days after admission Axial diffusion (A) and axial ADC map (B) show resolution in the previously seen extensive areas of diffusion restriction in the white matter of both cerebral hemispheres. ADC: apparent diffusion coefficient

During her stay in the PICU, she did not present with epileptic seizures and was extubated 19 days after improvement in the ventilatory parameters, removal of thoracic drainage, and once there was no requirement for hyperosmolar treatment or inotropic support. She presented with quadriparesis, hyperreflexia, and Babinski sign.

Outpatient evolution has been slow but favorable, with remarkable improvement in motor and language skills during the last year. Since discharge, she walks independently, reads, names things, repeats, and makes simple sentences. She has difficulties with learning and memory in the evolution.

## Discussion

Imaging evaluation in TBI plays an important role in daily clinical practice. A CT is the initial imaging modality for the diagnosis of primary injury (fractures, intracranial hemorrhage, or cerebral contusion), as it is a quick and relatively easy-to-access tool [8]. Several practice guidelines recommend its use for moderate and severe traumatic brain injuries [9,10].

An MRI has greater sensitivity for parenchymal lesions and is useful for the assessment of severity and prognosis in these patients; however, its high cost, acquisition times, and low availability maintain controversy over its recommendation as a routine imaging modality [10], with ranges of use between 25% and 70% of patients with severe traumatic brain injury [11]. There is also controversy over the appropriate time to request the exam, which ranges between three days and two weeks post-trauma [11].

The diffusion technique is part of the protocol at up to 93% of diagnostic centers [11]. This sequence measures the random movement of water molecules in the study tissue, showing diffusion restriction in pathologies such as cytotoxic edema and increased diffusion in pathologies such as vasogenic edema [12]. The volume and number of diffusion-restricted lesions are used as a predictor and correlate well with clinical practice scores for the evaluation of patients with traumatic brain injury [13-14].

Mechanisms for diffuse axonal injury (DAI) are rotational and rapid acceleration-deceleration forces on the brain that can lead to axonal white matter shearing and tearing [15]. Children are considered to be at risk of this injury due to the relatively decreased myelin content and higher water content in the pediatric brain [16].

Biochemical mechanisms involved in diffuse axonal injury include the caspase pathway conditioned by an increase in intracellular calcium, which can condition apoptosis and free radicals, initiating a cytotoxic cascade [17], with the consequent restriction in the diffusion technique, which is corroborated in experimental animal studies [18,19]. A pathway for cytotoxic edema includes glutamate, which is responsible for many neurologic functions, and leakage of the glutamate into the extracellular space results when there is a defect in the axonal membrane of the myelin sheaths [20].

Patients with TBI may present with various cerebral findings, such as hemorrhages, fractures, parenchymal contusions, and diffuse axonal injury, among others, depending on the strength of the trauma mechanism. Diffusion restriction, although it has been well described in patients with cerebral infarction as a sequel of a vascular lesion, there are very few reported cases of patients with white matter diffusion restriction secondary to cytotoxic edema of the parenchyma as it was presented in this patient. The first reported case dates from 2000 by Takayama [7], who observed areas of increased T2W signal and diffusion restriction in the cerebral peduncles and corpus callosum 12 days after the TBI in a 24-year-old patient with a clinic of slight right hemiparesis. These findings resolved spontaneously on a follow-up MRI six months later, with progressive improvement in neurological tests. In another case described by Yahya et al. [20] in an eight-year-old patient 11 days after TBI with diffusion restriction areas in the corpus callosum, periventricular white matter, and right centrum semiovale, 13 days later on follow-up MRI observed gliosis persistent in the splenium of the corpus callosum on T2WI and resolution of the areas of diffusion restriction. In our case, in the control MRI 10 days after admission, the areas of restriction diffusion were resolved with no significant gliosis remaining, and the patient came out of the coma.

Cytotoxic edema in these patients is explained by multiple hypotheses, but the exact pathophysiological mechanisms are still unknown. Yahya et al. postulate the release of glutamate in areas such as semioval centers and the corpus callosum; the absorption of this glutamate by axons could cause intramyelinic edema [20]. In the case of our patient, there was DAI with cytotoxic edema in the corpus callosum and white matter of both cerebral hemispheres.

There are very few studies that have reported this finding, and it is difficult to establish the prognosis of this type of patient and the future implications for their neurological performance. Zhen et al. studied the relationship between diffusion restriction and the duration of coma in patients with DAI in adult patients [13]. In our case, the patient was in a coma for eight days, and the MRI showed restriction diffusion in the exam performed during the coma (seven days after admission). Three days later, in the control MRI, no restriction diffusion was seen, and the patient recovered from the coma. It is recommended to carry out multidisciplinary interinstitutional work to obtain a greater number of cases of traumatic brain injury in children that present transient diffusion restriction in white matter with consequent evaluation of their clinical evolution and to establish the mechanism of their presentation and the prognosis of these lesions.

## Conclusions

Traumatic brain injury is a common condition in the pediatric population, frequently leading to high morbidity and potential neurological sequelae. Neuroimaging studies play a crucial role in prognostic disease; in addition to the known manifestations, there can be unusual presentations, such as transient diffusion restriction indicating cytotoxic edema; interestingly, these atypical findings are not associated with an unfavorable prognosis in the reported cases. Describing this presentation of DAI with transient diffusion restriction in cytotoxic edema contributes to medical knowledge in similar cases with a possible better clinical prognosis and fewer sequelae.
